# Massive *Ulva* Green Tides Caused by Inhibition of Biomass Allocation to Sporulation

**DOI:** 10.3390/plants10112482

**Published:** 2021-11-17

**Authors:** Masanori Hiraoka

**Affiliations:** Usa Marine Biological Institute, Kochi University, Inoshiri, Usa, Tosa, Kochi 781-1164, Japan; mhiraoka@kochi-u.ac.jp; Tel.: +81-88-856-0462

**Keywords:** biomass allocation, green tide, sporulation, *Ulva ohnoi*, *Ulva prolifera*, vegetative growth

## Abstract

The green seaweed *Ulva* spp. constitute major primary producers in marine coastal ecosystems. Some *Ulva* populations have declined in response to ocean warming, whereas others cause massive blooms as a floating form of large thalli mostly composed of uniform somatic cells even under high temperature conditions—a phenomenon called “green tide”. Such differences in population responses can be attributed to the fate of cells between alternative courses, somatic cell division (vegetative growth), and sporic cell division (spore production). In the present review, I attempt to link natural population dynamics to the findings of physiological in vitro research. Consequently, it is elucidated that the inhibition of biomass allocation to sporulation is an important key property for *Ulva* to cause a huge green tide.

## 1. Introduction

**Figure 1:**
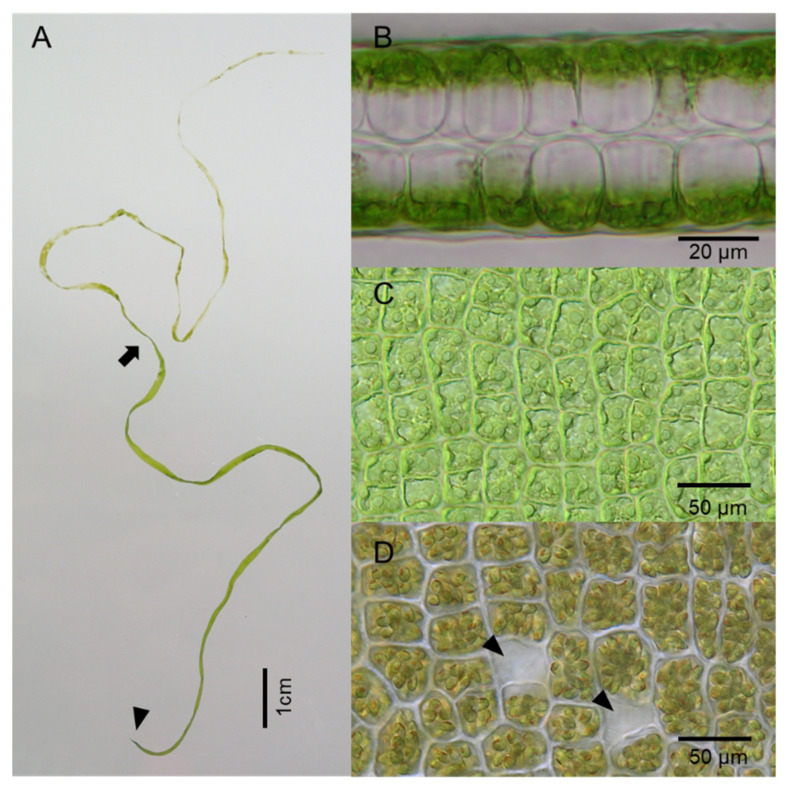
A living *Ulva* specimen (*U. aragoënsis*). (**A**) The developed thallus with sporulation in the upper part. Above the part indicated by the arrow, all the cells formed spores and some of them released spores. Arrowhead indicates a small holdfast; (**B**) Cross-section of the middle part of the thallus having a two-cell layered structure; (**C**) Surface view of somatic or blade cells in the vegetative state in the middle part of the thallus; (**D**) Surface view of the cells forming spores. Arrowheads indicate empty cells after spores were released.

*Ulva* (Ulvophyceae, Chlorophyta) or sea lettuce is the most abundant green seaweed and is ubiquitous in tropical and temperate coastal ecosystems around the world. The genus *Ulva* currently includes at least 85 taxonomically accepted species [1]. The thallus body is uniformly sheet-like, being two cells thick or tubular with a single cell layer, except for a very small holdfast part (Figure 1A,B). The generation time of *Ulva* is short, and its spores can develop into a thallus having the potential ability to produce spores again in 2–3 weeks. Spore formation occurs in the somatic cells which directly transform into sporangia (Figure 1C,D). The sporulation first occurs at the tip of the algal body and then sequentially occurs toward the bottom, several tens of spores per sporangia are released, and the empty sporangium are spontaneously detached from the thallus.

**Figure 2:**
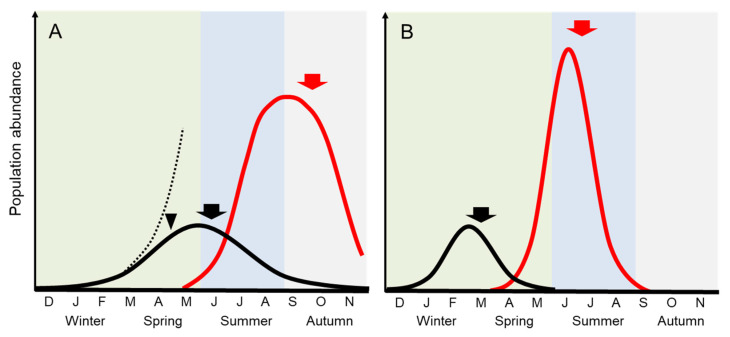
Comparison of seasonal abundance change between the attached populations and the green tides of *Ulva* in the temperate region. Arrowhead indicates the period when biomass increase is suppressed mainly by light limitation. Arrows show the period when allocation of biomass to sporulation begins to be greater than vegetative growth. (**A**) The attached *U. australis* modified from [9,12] (black line). Dotted line is a growth curve predicted in case of no light limitation. *Ulva ohnoi* green tide modified from [11] (red line); (**B**) Attached *U. prolifera* in brackish water modified from [13,14] (black line). The *U. prolifera* green tide in the Yellow Sea modified from [4,15] (red line).

A few *Ulva* species cause massive kilometer-scale blooms termed as green tides, and these have been recorded mainly around the industrialized coastlines of Europe, North America, and east Asia [2]. The world’s most extensive *Ulva* green tides have repeatedly occurred in the Yellow Sea since 2007 [3] or 2008 [4]. The causative green tide species can substantially increase their biomass in a free-floating state by increasing the size of the thalli and their fragments. Smetacek and Zingone [5] pointed out that this is crucial because it is the unattached forms that, by invading new space, are able to increase their nutrient supply, free themselves from competition for limited hard substrates, and avoid their many benthic grazers. As a result, the unattached forms can build up a large biomass, forming massive green tides. In general, most *Ulva* spp. grow when their motile spores settle on the substrate. The attached populations in temperate regions are regularly present in early spring, increase to a maximum in late spring, and rapidly decrease through summer, showing a unimodal pattern of biomass fluctuation (Figure 2A) [6,7,8,9]. The summer decline of the attached populations occurs markedly at 20–25 °C. However, green tide species peak in biomass in the summer [10] and occasionally continue to grow [11]. Such a temporal difference of thallus growth pattern between attached populations and floating green tide populations has not received much attention in the literature. I consider that the species-specific differences of ecophysiological characteristics are a crucial key in determining if an algal species causes an excessive bloom or not.

In various previous publications, it has been explained that *Ulva* green tides are a symptom resulting from eutrophication [16]. Indeed, the supply of dissolved inorganic nitrogen (N) and phosphorus (P) is required to sustain *Ulva* thallus growth. However, as an example of green tides in Tokyo Bay, the scale of *Ulva* blooms has expanded despite a significant decrease in N and P concentrations year by year [17]. In this case, a subtropical species, *U. ohnoi*, unintentionally introduced and excessively grew as unattached form without the summer decline. In the Yellow Sea, free-floating *Ulva* populations during the early stage of green tides in spring include four or more *Ulva* species, but only one species, *U. prolifera*, continuously expand, resulting in monospecific spectacular blooms in summer [18,19]. All the other species do not seem to be able to continue vegetative growth while enduring the high temperatures from spring to summer, even though they sympatrically experience the same eutrophic conditions in the Yellow Sea. These examples of *U. ohnoi* and *U. prolifera* indicate that when specific species, having a continuous growth ability in high temperatures, encounter the minimum nutrient conditions for sustaining its vegetative growth, a huge bloom can occur.

In this review, I compare the *Ulva* biomass fluctuation patterns between attached populations and green tide populations and explain which ecophysiological properties of a species or strain is essential for causing green tides. In addition, I will focus on the mechanism of switching between the vegetative growth and sporulation of *Ulva* cells. By relating field observations and laboratory experiments, I attempt to more comprehensively examine the relationships between the cells, individuals, and populations underlying the mechanism of the development of green tides.

## 2. Attached Population Dynamics

### 2.1. Population Fluctuation Follows Individual Size Fluctuation

In the temperate coastal zone, the biomass of attached *Ulva* populations fluctuates seasonally according to the periodic fluctuation of the water temperature. Many temperate species such as *U. rigida*, *U. lactuca*, and *U. australis* (syn. *U. pertusa*) show a regular fluctuation pattern in which the attached biomass increases from winter to spring and declines during high temperatures from summer to autumn, as described in Figure 2A [6,7,8,9,12,20,21]. The biomass peaks shift to later in the year in cold regions at high latitudes [22]. This review will progress the story about the temperate populations. There has been detailed demographic research study on the attached population of *U. australis* over a period of three consecutive years [9]. It demonstrated that the seasonal fluctuations of the *Ulva* population synchronize with those of the thallus size rather than with changes in the density of thallus individuals. This indicates that biomass fluctuations of *Ulva* population are attributed to that of well-developed thallus individuals.

### 2.2. Increase Phase

*Ulva* with a simple multicellular body perform ‘diffuse growth’ in which cell divisions can occur more or less throughout the tissues of the organism [23]. The somatic cells divide synchronously in standardized conditions once a day [24]. Therefore, the *Ulva* thalli are capable of exponential growth, displaying extremely high growth rates. In fact, a daily rate of over fourfold in *U. meridionalis* in the culture experiment has been reported as the highest growth rate ever reported for multicellular autotrophic plants. In the same paper, a strain isolated from the attached *U. prolifera* population was also revealed to display two-fold growth rate per day [25]. The high exponential growth of *Ulva* spp. generally occurs in high temperatures of 20–30 °C in suitable light and nutrient conditions, as described below in Section 3. If such high growth continues in the sea from spring to summer with the optimum high temperatures, a bloom would occur explosively. However, in the attached population, the rapid biomass increase is suppressed mainly by light limitation caused by self-shading as density increases (Figure 2A). Although light limitation is an inevitable suppression factor, the population is also negatively affected by some irregular factors of low salinities by precipitation and herbivory by benthic organisms such as snails and sea hares. Their inhibitory effects occur because the population is attached to the substrate.

### 2.3. Decline Phase

As the water temperature rises over 20 °C, *Ulva* thalli are highly promoted to produce and release spores. The allocation to sporulation in thalli of the attached *Ulva* populations has been observed to be significantly larger during warmer months [12,20]. Niesenbaum [20] described “the seasonality of reproduction, and changes in the abundance of total biomass and reproductive biomass during the reproductive season could have been a function of temperature. The sharp decline of total biomass in early August, and its low rate of recovery through August and September were probably due to temperature effects on growth and reproduction”. Furthermore as “When temperature reached seasonal highs, allocation of biomass to the formation and release of swarmers (spores) was greatest, while the rate of vegetative replacement diminished as temperatures first became suboptimal and then inhibitory for growth. This could explain the increases in percent reproductive tissue during August and September”. These findings are essential for understanding the *Ulva* biomass fluctuation. However, so far the allocation of biomass to sporulation in the decline phase of *Ulva* populations has been almost overlooked. Practically, only the intrinsic traits involved in the increase phase, such as high growth rates or multiple reproduction modes, have been highlighted [3,26]. Recently, a few works examined the decline phase of the *U. prolifera* green tide in the Yellow Sea [10]. However, little coverage has been given to the allocation to sporulation.

### 2.4. Disappearance Phase

After the decline phase, *Ulva* thalli almost disappear in the autumn. In this disappearance phase, although evidence has not been provided from field research yet, small individual thalli of less than a few centimeters in size could release spores and disappear, whereby their settled spores grow up fast to small thalli and release spores again in the early developmental stage. This fast generation alternation may occur until the water temperature drops below 20 °C in temperate species. These predictions are derived from culture work in the laboratory as described next.

## 3. Individual Size Determined by Vegetative Growth and Sporulation Decay

**Figure 3:**
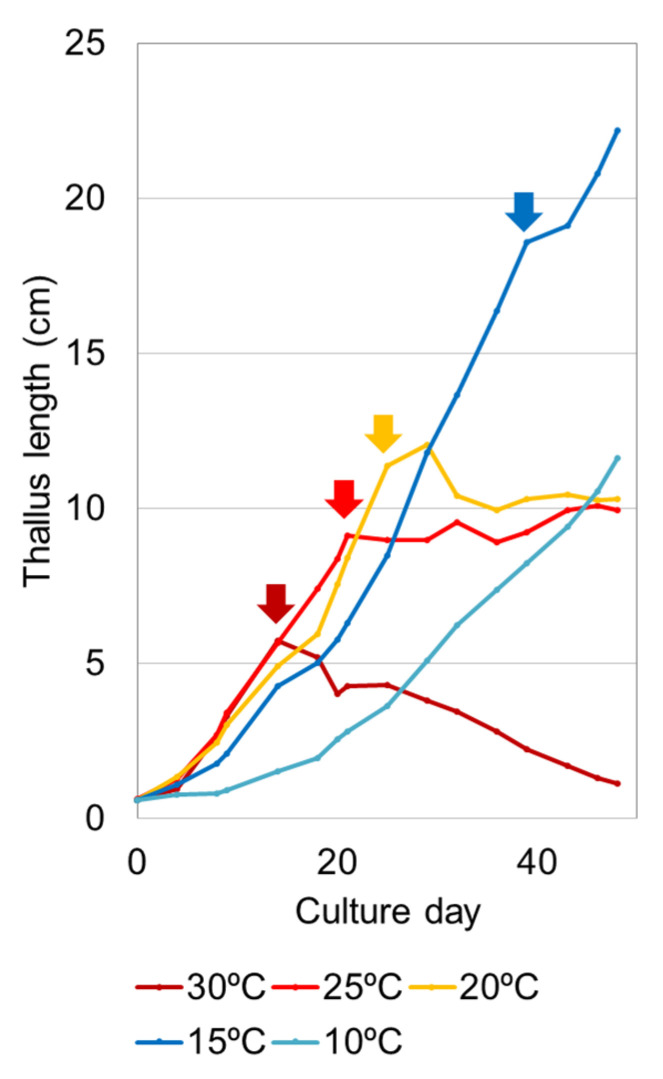
Change of averaged thallus length of *Ulva prolifera* strain isolated from the attached population in the Yoshino River estuary, Japan, cultured at different temperatures. Each arrow indicates the day when sporulation first occurred. After that, sporulation occurred repeatedly. Only at 10 °C, no sporulation occurred. This figure was redrawn based on the data from [27].

Culture experiments using temperature-controlled incubators have confirmed that higher temperatures promote sporulation decay [27]. An asexual variant of *U. prolifera* originally isolated from the attached population in brackish water and its clonal offspring thalli were tested (Figure 3). According to this study, their growth rates increase as the temperature rises to 25 °C. However, sporulation at the apical part of the thallus occurs earlier as the temperature rises, and the amount of sporulation decay increases. At 30 °C after sporulation first occurs, the thalli repeatedly produce spores and as a whole continue to decrease, resulting in the disappearance in one and half months of culture. At 20 °C and 25 °C, the vegetative growth increment and the amount of decay due to sporulation are balanced, and the total length of the thalli cannot extend from about 10 cm. At 15 °C, because the vegetative growth exceeds the small amount of sporulation, the thalli continue to grow larger. At the low temperature of 10 °C, sporulation does not occur, and the thalli continue to grow slowly. From these findings, individual thallus mass (M) can be expressed by the two factors of vegetative growth (G) and the amount allocated to sporulation (S) as M = G − S. As the *U. prolifera* strain has the intrinsic trait of S ≥ G at ≥20 °C after the first sporulation, M becomes constant or decreases over 20 °C.

## 4. Inhibition of Sporulation Leads to Green Tides

### 4.1. Ulva ohnoi Green Tide

Floating thalli which are free from the attached substrate can spread moderately and receive sufficient light. If the floating thalli acquires the property of inhibiting sporulation even at high temperatures, they would make a massive green tide. Here, the intrinsic trait to cause a large-scale green tide is expressed as S < G at ≥20 °C. Then, as S is nearly zero and G is usually the maximum growth rate that the *Ulva* species can attain, M increases exponentially. In addition to the high-temperature growth property, the structural feature of easily producing floating thalli and their fragments also promotes the magnification of the green tides. *Ulva ohnoi* is a typical species with these characteristics. When this species was reported as a new green tide-forming species, it was taxonomically described as having a large, thin, and fragile blade thallus easily torn into floating fragments in the diagnosis [28]. The field observation of the *U. ohnoi* green tide was first made in Tosa Bay, southwestern Japan, for two years [11]. It shows that the thallus fragments grew rapidly at over a five-fold growth rate per week as the summer water temperature rose to 28–30 °C, reaching an average length of about 50 cm, a maximum length of >1 m, and the largest biomass attained approximately 1 kg fresh mass m^−2^ in August. In the decline phase of the green tides, a small amount of sporulation occurred in June-August, but half and more parts of the well-developed blades frequently formed and released spores in October, resulting in the population decline (Figure 2A). The remarkable difference compared to the temperate attached population is that *U. ohnoi* continues to grow with no or little biomass allocation to sporulation during the period of the summer decline. The properties are summarized below.

1. Sporulation which is generally promoted at high temperatures is suppressed.
2. At high temperatures, relatively high growth ability is exhibited.
3. Free-floating thallus fragments are easily formed.

Of these, 1 is particularly important for the occurrence of green tides. If the species does not produce spores and does not reduce its total mass, the green tide biomass gradually develops even if the growth is not so fast. A fragmentation culture method available for investigating the likelihood of sporulation in *Ulva* has been presented [29]. Applying the method, sporulation can be induced in 2 to 3 days on *Ulva* thallus tissue collected from the attached population [30]. By the same method, however, the thallus blades of *U. ohnoi* and the other *Ulva* spp. sampled from the massive green tides showed no or a very low frequency of induced sporulation or took a longer time to sporulation [31]. Before *U. ohnoi* was taxonomically differentiated as a new species, this species blooming in Ohmura Bay, southwestern Japan, had been identified as a sterile mutant of *U. pertusa* (now *U. australis*) [32] and it is still believed to be so [33]. Migita [32] showed that 1 cm^2^ thallus fragments of his *U. ohnoi* strain displayed the maximum growth rate of two-fold growth rate per 2 days in laboratory experiments at 20 °C, and then transplanted into an outdoor tank and grew up to larger than 1 m^2^ in 2 months without any sporulation, while all the fragments of more than 10 wild *U. australis* thalli formed spores in the same culture conditions, resulting in the disappearance of the thallus. These results indicate that bloom-forming species have a physiological property of being less prone to sporulate, or they do not sporulate.

*Ulva ohnoi* distribute mainly in the subtropical region and are adapted to high temperatures. Therefore, it has spread to the temperate area and outbreaks in the summer. The spread of *U. ohnoi* has been increasingly reported from various regions [34,35,36]. Due to global warming, *U. ohnoi* may spread further into higher latitudes and cause green tides. However, in Tosa Bay, where the *U. ohnoi* green tide was first reported, this species has recently decreased sharply and instead, *U. reticulata*, which has a distribution centered in more tropical waters, has begun to increase [37]. This example suggests that each *Ulva* species has a temperature range that balances the vegetative growth and sporulation, and that individual thallus growth, or population growth, may not be possible if the temperature limit is exceeded even by a few degrees.

### 4.2. Ulva prolifera Green Tide

The *U. prolifera* green tides in the Yellow Sea regularly reach their biomass peak during June and July in summer (Figure 2B) [4,15]. The earliest free-floating *Ulva* patches are found in the coastal areas of the southern Yellow Sea from mid-April to early May [3,18]. These patches originally appear off the nearby rafts for purple laver (*Neopyropia yezoensis*) aquaculture, for which the coverage area is approximately 4.1 × 10^4^ ha [3]. Annually, approximately 6500 t of the *Ulva* mass has been estimated to be released as macroalgal waste from mid-April to late-May after cleaning the *Neopyropia* aquaculture facilities [38]. In this early stage, the patches include multiple *Ulva* spp. such as *U. linza*, *U. compressa*, and *U. aragoënsis* (=*U. flexuosa* in [18,19]). However, the free-floating *Ulva* complexes move northward, associated with the seasonal monsoons and ocean currents, rapidly develop into long large bands ranging from hundreds of meters to tens of kilometers in the open sea area in late May [3], and then massive green tides are dominated by a single species, *U. prolifera* [18,19,39]. Only this species explosively grows, while the other species disappear over 20 °C in early summer. This suggests that the bloom-forming *U. prolifera* can continuously grow inhibiting allocation to sporulation in high temperatures, thereby differing from the other *Ulva* spp. in this region. Supporting this finding, the culture work showed that a few centimeters of the bloom-forming *U. prolifera* fragment can grow to more than 50 cm in length without sporulation at 20 °C (cf. Figure 12 in [40]). This growth characteristic is obviously different from that of the *U. prolifera* strain from the attached population, which cannot grow over 10 cm at ≥20 °C (Figure 3). However, the bloom-forming *U. prolifera* seems to allocate its biomass to sporulation around 25 °C, because it was observed that the green tide population began to decline at 25 °C from July to August [10].

**Figure 4:**
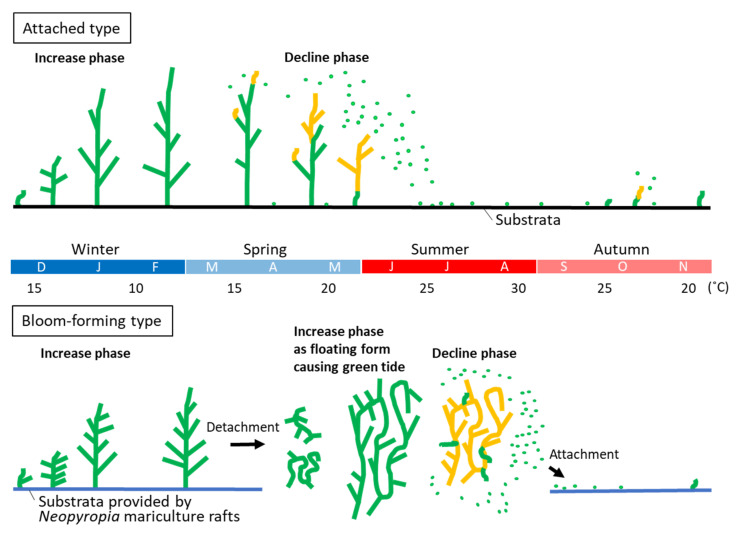
Comparison of seasonal change of thallus state between the attached type, *Ulva prolifera* subsp. *prolifera*, and the bloom-forming type, *U. prolifera* subsp. *qingdaoensis*. Green and orange lines of thallus image show vegetative state and sporulating state, respectively. Green dots indicate microscopic propagules or spores released from the sporulated thalli. The two types have significantly different timings of the decline phase.

Different from the bloom-forming type, the common attached type of *U. prolifera* form abundant populations on the substrate in brackish waters such as river estuaries [41,42]. Seasonal fluctuations of the largest attached *U. prolifera* population in Japan have been described in detail (Figure 2B) [13,14]. The biomass and thallus length of the attached population regularly reach their maximum from January to March and then disappear by July. Although natural *U. prolifera* mats develop in the Chinese coast located in the south of the Yellow Sea and are harvested as edible biomass, the peak harvest is also from January to March [43], which is consistent with the fluctuation pattern of the populations in Japan. Although the attached *U. prolifera* has been used as an expensive macroalgal ingredient for Japanese dishes for a long time, its harvest has become a ‘winter’ tradition [13]. However, in line with recent ocean warming, the annual yield is declining [44]. It can be explained that the biomass decrease of the attached population is due to the shortening of the period when the water temperature falls below 20 °C. The most significant difference of the ecophysiological property between the bloom-forming type and the attached type in *U. prolifera* is whether they can vegetatively grow inhibiting allocation to sporulation around 20 °C or not. The seasonal difference is described in Figure 4.

Although the issue of the massive green tide in China received a great deal of attention in 2008 [45], immediately after that in some research papers the bloom-forming type and the attached type were not distinguished, and both were confused because they formed a monophyletic clade together with the most closely related species, *U. linza*, by molecular analysis using the nuclear-encoded rDNA internal transcribed spacer (ITS) region, which is commonly used for *Ulva* species identification. However, studies of culture, hybridization, and phylogenetic analysis using a higher resolution DNA marker (5S rDNA spacer region) revealed that the bloom-forming type can cross with the attached type without any reproductive boundary, which was confirmed to be conspecific, but there are several genetic and ecophysiological differentiations (Table 1). Consistently, the other genetic analyses using inter-simple sequence repeat markers and a sequence-characterized amplified region marker indicated that the bloom-forming type is a unique ecotype of *U. prolifera*, genetically distinct from the attached types along the Chinese coast [46].

From the phylogenetic analyses of *U. linza* and *U. prolifera* using the 5S sequence, it has been suggested that *U. prolifera* had adapted to brackish water and recently evolutionarily separated from marine *U. linza* [42]. Furthermore, crossing tests suggested that the bloom-forming type of *U. prolifera* completed the speciation from *U. linza* via intermediate brackish *U. prolifera* (=the attached type) because there is still a partial compatibility between the common brackish *U. prolifera* and *U. linza*, but a complete reproductive barrier exists between the bloom-forming *U. prolifera* and *U. linza* [48]. The brackish *U. prolifera* contains many regional populations that have genetically differentiated [42]. One of them may have acquired the ability to suppress sporulation and continue vegetative growth even at 20 °C or higher, resulting in creating the *U. prolifera* subsp. *qingdaoensis* that cause green tides. Interestingly, the *U. prolifera* subsp. *qingdaoensis* is characterized by a densely branching morphology (Table 1). This feature may facilitate the production and dispersal of large numbers of floating thallus fragments.

In contrast to *U. ohnoi*, widely reported from various regions, the occurrence of *U. prolifera* green tides has been limited to the Yellow Sea only. The special *U. prolifera* population unique to this region is fostered in the vast *Neopyropia* farm as its nursery bed, and is supplied annually as a large amount of floating mass [3,38]. The world’s largest green tide appears to be caused by such a very special production cycle supported by the aquaculture activities.

## 5. Mechanism of Sporulation

The multicellular body of *Ulva* is composed of mostly uniform blade cells (or somatic cells) except for a small number of rhizoid cells forming a small holdfast (Figure 1). Therefore, the allocation of the individual thallus tissue to vegetative growth and sporulation is almost attributed to the blade cell fate between the alternative courses, somatic cell division, and sporic cell division. Nordby [29] hypothesized that the cell fate is controlled by changes in the concentration of sporulation inhibitors. This inhibitor hypothesis inferred that the double-layered structure of the *Ulva* thallus (Figure 1B) could be responsible for maintaining a sufficient concentration of the sporulation inhibitor during vegetative growth. Supporting that, Stratmann et al. [49] revealed that the *Ulva* thallus produces at least two kinds of the sporulation inhibitor, one of which is a glycoprotein ‘Sporulation inhibitor-1′ (SI-1), and the other is a nonprotein of very low molecular mass (SI-2). The SI-1 is present in the cell wall of *Ulva* cells and appears to be secreted extracellularly. The SI-2 is in the inner space between the two blade cell layers. The excretion of the SI-1 decreases with maturation of the thallus, whereas the overall concentration of SI-2 in the thallus stays constant throughout the life cycle. The SI-2 affects different *Ulva* species whereas the SI-1 is species-specific. Although such characteristics of the inhibitors have been shown, their molecular structures have not yet been identified.

The fragmentation culture method can synchronously induce sporulation in *Ulva* thallus tissue within 48 h, when fragmented single-layered thalli are transferred to fresh medium at a low density of fragments and cultured in optimal conditions [29]. It is explained by the inhibitor hypothesis that the inhibitors leak out from the circumference of fragmented thalli and the somatic cells that sense the decrease in the concentration of the inhibitors go to sporulation. As already mentioned, the bloom-forming *Ulva* spp. hardly, or do not, allocate the somatic cells to sporulation, even in high temperatures. Considering the inhibitor hypothesis, it is possibly thought that the bloom-forming species produce a large amount of the inhibitory substances or have an inhibitor-sensing system in which it is hardly relieved from the inhibition. The entire genome of *Ulva* has already been announced [50]. Therefore, if the inhibitors are structurally identified, it is expected that the elucidation of the switching mechanism between the somatic cell division and the sporic cell division will be achieved.

## 6. Conclusions and Perspective

By comparing attached populations and green tide populations, it became clear that the bloom-forming species continue to grow vegetatively with almost no spore formation even at high temperatures. However, few field surveys have been conducted on the process of the decline of green tide populations from the viewpoint of the allocation to sporulation, and future investigations are required.

Though the sporulation inhibitors were partially characterized in 1996, their molecular structures have not yet been determined. These substances are the key to determining the fate of somatic cell division or sporic cell division. It is highly possible that the causative species of the green tide have different reaction systems involving the sporulation inhibitors. Such research studies are more likely to detect minor differences when comparing taxa containing blooming strains and non-blooming strains within the same species. In that sense, *U. prolifera* would be excellent experimental material.

*Ulva* is a promising organism for carbon dioxide fixation and bioproduct production due to its high productivity [51,52]. Understanding the allocation system of vegetative growth and sporulation decay will enable greater control of biomass production and will contribute to the development of the bioeconomy.

## Figures and Tables

**Table 1:** Comparison between the bloom-forming type and the attached type in *Ulva prolifera*.

	Bloom-Forming Type	Attached Type
Subspecies name	*Ulva prolifera* subsp. *qingdaoensis*	*Ulva prolifera* subsp. *prolifera*
Habitat/Distribution	Offshore and coastal waters in the Yellow Sea	Brackish and estuarine waters in cold–temperate regions
Season for maximum population abundance	Early summer	Winter to early spring
Temperature at which thallus growth turns to zero or negative	Around 25 °C	Around 20 °C
Branch density of cultured thalli	>100 per 1 cm of main stem	0.4–14 per 1 cm of main stem ^1^
Life cycle	Sexual	Sexual or obligate asexual by special spore (zoosporoid)
Crossing affinity to *Ulva linza*	Complete incompatibility	Partial gamete incompatibility
5S sequence	5S-A type or 5S-B type ^2^	A large number of various types ^3^ different from 5S-A and 5S-B types

^1^ Data from the type locality population [40]; ^2^ The two types reported by [18]; ^3^ Thirty-one types reported by [42] and several more [19,43,47].
